# Validation of housekeeping genes for quantitative real-time PCR in in-vivo and in-vitro models of cerebral ischaemia

**DOI:** 10.1186/1471-2199-10-57

**Published:** 2009-06-16

**Authors:** Carme Gubern, Olivia Hurtado, Rocío Rodríguez, Jesús R Morales, Víctor G Romera, María A Moro, Ignacio Lizasoain, Joaquín Serena, Judith Mallolas

**Affiliations:** 1 Servei de Neurologia, Fundació Privada Institut d'Investigació Biomèdica de Girona Dr. Josep Trueta (IdIBGi), Hospital Universitari de Girona Dr. Josep Trueta, Girona, Spain; 2 Departamento de Farmacología, Facultad de Medicina, Universidad Complutense de Madrid (UCM), Madrid, Spain

## Abstract

**Background:**

Studies of gene expression in experimental cerebral ischaemia models can contribute to understanding the pathophysiology of brain ischaemia and to identifying prognostic markers and potential therapeutic targets. The normalization of relative qRT-PCR data using a suitable reference gene is a crucial prerequisite for obtaining reliable conclusions. No validated housekeeping genes have been reported for the relative quantification of the mRNA expression profile activated in in-vitro ischaemic conditions, whereas for the in-vivo model different reference genes have been used.

The present study aims to determine the expression stability of ten housekeeping genes (Gapdh, β2m, Hprt, Ppia, Rpl13a, Oaz1, 18S rRNA, Gusb, Ywhaz and Sdha) to establish their suitability as control genes for in-vitro and in-vivo cerebral ischaemia models.

**Results:**

The expression stability of the candidate reference genes was evaluated using the 2^-ΔC'T ^method and ANOVA followed by Dunnett's test. For the in-vitro model using primary cultures of rat astrocytes, all genes analysed except for Rpl13a and Sdha were found to have significantly different levels of mRNA expression. These different levels were also found in the case of the in-vivo model of pMCAO in rats except for Hprt, Sdha and Ywhaz mRNA, where the expression did not vary. Sdha and Ywhaz were identified by geNorm and NormFinder as the two most stable genes.

**Conclusion:**

We have validated endogenous control genes for qRT-PCR analysis of gene expression in in-vitro and in-vivo cerebral ischaemia models. For normalization purposes, Rpl13a and Sdha are found to be the most suitable genes for the in-vitro model and Sdha and Ywhaz for the in-vivo model. Genes previously used as housekeeping genes for the in-vivo model in the literature were not validated as good control genes in the present study, showing the need for careful evaluation for each new experimental setup.

## Background

Oxygen-glucose deprivation (OGD) and middle cerebral artery occlusion (MCAO) are accepted as in-vitro and in-vivo cerebral ischaemia models [1-4]. The permanent MCAO (pMCAO) focal ischaemia model is widely used in stroke research and is helpful in elucidating physiopathological causes and identifying prognostic markers and potential therapeutic targets [5-9].

Real-time polymerase chain reaction (RT-PCR) is a powerful technique for gene expression studies due to its high sensitivity, specificity and broad quantification range for high throughput and accurate expression profiling of selected genes [10]. The data obtained by quantitative RT-PCR (qRT-PCR) is typically normalised with an internal control, often referred to as a housekeeping gene. Up to now, several mathematical methods, such as NormFinder [11], geNorm [12] and 2^-ΔC'T ^method [13], have been developed to analyse the variability of the expression of candidate housekeeping genes. The ideal housekeeping gene for qRT-PCR would be one whose mRNA is consistently expressed at the same level in all samples under investigation, regardless of tissue type, disease state, medication or experimental conditions, and would have expression levels comparable to that of the target [14,15]. However, no ideal reference gene has yet been discovered and it is reasonable to suppose that such a gene may not exist. Although different genes have been used, several previous studies have shown that the suitability of a particular reference gene depends on the insult, lesion and experimental conditions applied to the cell culture or experimental animal [16,17]. Therefore, careful evaluation and validation of control genes is required prior to using them in each individual case to avoid possible inaccuracies stemming from the use of an unsuitable reference gene [14,16,18-20].

In addition, no validated housekeeping genes have been reported for the relative quantification of the mRNA expression profile activated in in-vitro ischaemic conditions. With regards to the in-vivo model (pMCAO), *Glyceraldehyde-3-phosphate dehydrogenase *(Gapdh) [16,21,22], *Peptidyl-prolyl cis-trans isomerasa *(Ppia) [21-23], *Ribosomal Protein L13A *(Rpl13a)[23] and *Hypoxanthine phosphoribosyltransferase *(Hprt) [16] have previously been accepted as control genes. The aim of this study was to confirm the validity of these reference genes in our pMCAO samples and to test their usefulness as endogenous controls in the in-vitro model using primary cultures of rat astrocytes. Furthermore, we aimed to analyse the expression stability of other reference genes that have been used, *Beta-2 microglobulin *(β2m) and *18S rRNA *[24], *Glucoronidase B *(Gusb) [25], *Succinate dehydrogenase complex, subunit A *(Sdha) [14,25,26], *Tyrosine 3-monooxygenase/tryptophan 5-monooxygenase activation protein, zeta polypeptide *(Ywhaz) [14,26] and a new candidate gene for the in-vivo model, *Ornithine decarboxylase antizyme 1 *(Oaz1), which has been hypothesised as being suitable in experimental conditions involving high protein turn-over [10].

## Results

### Evaluation and validation of selected candidate housekeeping genes in an in-vitro ischaemia model

**Figure 1:**
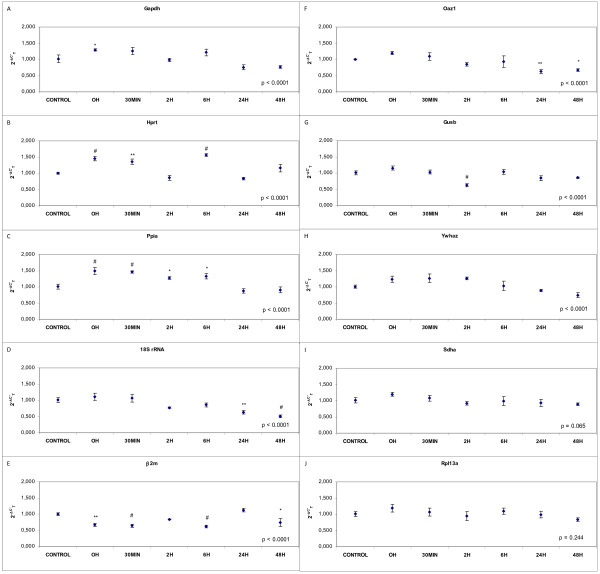
**Effects of OGD in astrocyte expression of housekeeping genes: (A) Gapdh, (B) Hprt, (C) Ppia, (D) 18S rRNA, (E) β2m, (F) Oaz1, (G) Gusb, (H) Ywhaz, (I) Sdha and (J) Rpl13a**. Fold change in gene expression analysed by the 2^-ΔC'T ^(see methods for details). Data are mean ± S.E.M., n = 3: * p < 0.05, ** p < 0.01, ^# ^p < 0.0001 vs. control.

The results of gene expression of Gapdh, Hprt, Ppia, 18S rRNA, β2m, Oaz1, Gusb, Ywhaz, Sdha and Rpl13a from astrocytes exposed to OGD during 3 hours and collected at 0 minutes, 30 minutes, 2, 6, 24 and 48 hours after reperfusion are shown in Figure 1.

The expression of Gapdh was found to have significant fluctuations in the levels of mRNA (p < 0.0001) increasing significantly at 0 h after reperfusion (p < 0.05) whereas no significant differences were observed at other points (Figure 1A). The expression of Hprt, in comparison to the control group, was found to fluctuate significantly in all the groups as a whole (p < 0.0001). This fluctuation was a significant increase at 0 min (p < 0.0001), 30 min (p < 0.01) and 6 h after reperfusion (p < 0.0001) (Figure 1B). Significant differences in the expression of Ppia were found between time groups (p < 0.0001). Levels increased at 0 and 30 min (p < 0.0001 in both cases) and at 2 h and 6 h (p < 0.05 in both cases) after reperfusion in comparison with the control group (Figure 1C). The 18S rRNA mRNA showed significant level fluctuations (p < 0.0001). Levels decreased at 24 h (p < 0.01) and 48 h (p < 0.0001) after reperfusion (Figure 1D). Changes in β2m mRNA levels were found to be significant between time groups (p < 0.0001). β2m expression decreased at 0 min (p < 0.01), 30 min and 6 h (p < 0.0001 in both cases) and 48 h (p < 0.05) in comparison with the control group (Figure 1E). Oaz1 also showed significant differences in expression between groups (p < 0.0001). The expression was stable at the first four analysis times but decreased significantly at 24 h (p < 0.01) and 48 h (p < 0.05) (Figure 1F). Significant fluctuations in the mRNA expression of Gusb were found (p < 0.0001) decreasing at 2 h after reperfusion (p < 0.0001) (Figure 1G). The expression of Ywhaz was found to have significant fluctuations in mRNA levels (p < 0.0001) (Figure 1H). Analysis of the same samples with a specific gene expression assay for Sdha and Rpl13a did not reveal any significant differences in expressions at the different time points when analysis was made (p = 0.065 and p = 0.244, respectively) (Figure 1I and 1J).

### Evaluation and validation of selected candidate housekeeping genes in an in-vivo cerebral ischaemia model

**Figure 2:**
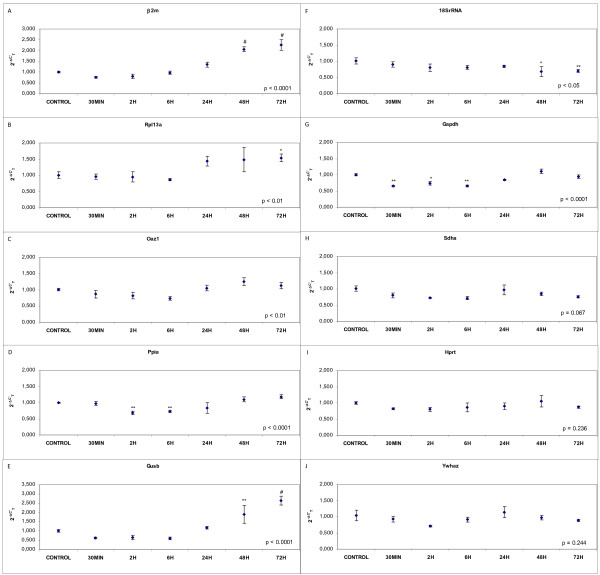
**Effects of pMCAO in expression of housekeeping genes: (A) β2m, (B) Rpl13a, (C) Oaz1, (D) Ppia, (E) Gusb, (F) 18SrRNA, (G) Gapdh, (H) Sdha, (I) Hprt and (J) Ywhaz**. Fold change in gene expression analysed by the 2^-ΔC'T ^(see methods for details). Data are mean ± S.E.M., n = 6: * p < 0.05, ** p < 0.01, ^# ^p < 0.0001 vs. control.

The gene expression of candidate housekeeping genes, β2m, Rpl13a, Oaz1, Gapdh, Ppia, Gusb, 18SrRNA, Sdha, Ywhaz and Hprt, in cerebral cortex samples collected at 30 minutes and 2, 6, 24, 48 and 72 hours after pMCAO is shown in Figure 2.

β2m expression presented a significant increase at 48 and 72 h (p < 0.0001 in both cases) and was significantly different between groups (p < 0.0001) (Figure 2A). The expression of Rpl13a increased 72 h after pMCAO (p < 0.05) with a significant fluctuation between groups (p < 0.01) (Figure 2B). The level of mRNA variation of Oaz1 was found to be significant for all the time groups taken as a whole (p < 0.01) (Figure 2C). Significant differences were found between groups for the expression of Ppia (p < 0.0001) with mRNA decreasing significantly at 2 and 6 h (p < 0.01 in both cases) after pMCAO (Figure 2D). The expression of Gusb was found to fluctuate significantly between the different test points (p < 0.0001). At 48 h and 72 h, levels of Gusb mRNA had increased (p < 0.01 and p < 0.0001 respectively) (Figure 2E). The level of the mRNA of 18S rRNA showed significant fluctuation (p < 0.05) decreasing significantly at 48 h (p < 0.05) and 72 h (p < 0.01) (Figure 2F). Changes in Gapdh mRNA levels were found to be significant between time groups (p < 0.0001). Gapdh expression decreased at 30 min (p < 0.01), 2 h (p < 0.05) and 6 h (p < 0.01) in comparison with the control group (Figure 2G). mRNA expression was invariable under in-vivo ischaemic brain conditions for the Sdha (p = 0.067), Hprt (p = 0.236) and Ywhaz (p = 0.244) genes (Figure 2H–2J).

**Figure 3:**
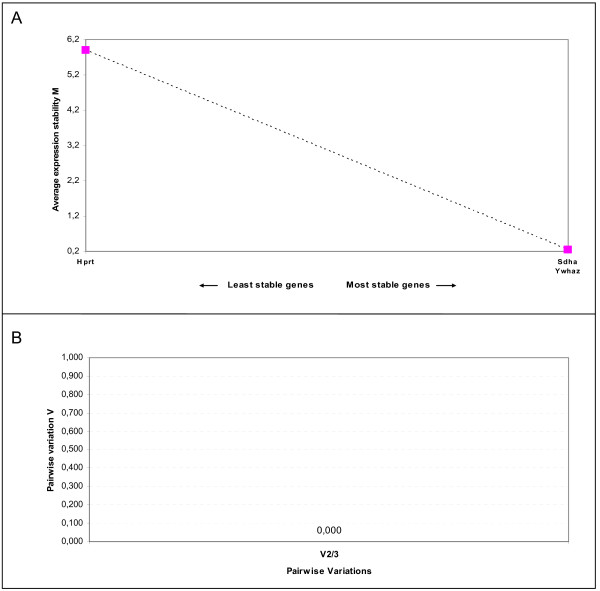
**Gene expression stability and determination of the optimum number of genes for normalization of the candidate reference genes for the in-vivo ischaemia model using geNorm analysis**. (A) Expression stability plot showing average expression stability values M. (B) Pairwise variation analysis to determine the optimal number of reference genes for use in RT qPCR data normalization. The use of the two most stable genes is sufficient for accurate normalization (cutoff 0.15 according to [12])

The stability of these three genes was evaluated using geNorm and NormFinder algorithms. GeNorm identified Ywhaz and Sdha as the most stable pairwise combination of reference genes for the in-vivo ischaemia model (Figure 3A). Figure 3B shows the pairwise variation value (V) between two sequential normalization factors containing an increasing number of genes. As we only analysed three genes, the figure shows the pairwise variation value of V2/3 (0.000). According to Vandesompele et al. [12], the ideal pairwise variation value is less than 0.15 and therefore two genes, in our case Ywhaz and Sdha, are enough for normalization. NormFinder identified Ywhaz as the most stable gene with a stability value of 0.023 and, as with geNorm, the best two genes for normalization were Ywhaz and Sdha with a stability value of 0.022 (Table 1).

**Table 1:** Stability values of housekeeping genes obtained by NormFinder

**Ranking**	**Gene name**	**Stability value**
**1**	**Ywhaz**	**0.023**
2	Sdha	0.034
3	Hprt	0.066

**Best combination of two genes**	**Sdha and Ywhaz**	**0.022**

## Discussion

The selection of the housekeeping gene is crucial to the normalization of quantitative gene expression results. This is even more important in those settings in which gene expression is severely affected, as after cerebral ischaemia.

No validated housekeeping genes have been reported for the relative quantification of the mRNA expression profile activated in in-vitro ischaemic conditions. Our present study discards the use of Gapdh, Ppia, β2m, 18S rRNA, Hprt, Oaz1, Gusb and Ywhaz as reference genes and finds Rpl13a and Sdha to be the best control genes for the in-vitro model in the conditions we have tested.

With regards to the pMCAO model, four studies have reported the suitability of several reference genes for this ischaemia model. In Table 2 we summarise the methodologies employed and results obtained both for previously published and the present studies. Earlier investigations only tested from two to four candidate control genes finding invariable expressions for Rpl13a, Hprt, Ppia and Gapdh genes. In the case of Gapdh, results were contradictory: three studies validated the gene [16,21,22] whereas another rejected it [23]. Our results show that Gapdh, Rpl13a and Ppia together with other candidate genes, namely β2m, Gusb, 18S rRNA and Oaz1, are not appropriate as housekeeping genes for the pMCAO model. As previously reported by Meldgaard et al. when using the pMCAO model in mice [16], we have found Hprt to be a suitable housekeeping gene for the in-vivo ischaemia model. Moreover we have identified two new housekeeping genes for this model, Sdha and Ywhaz, which have not been previously analysed. According to the results obtained with the NormFinder and geNorm algorithms, these two genes are more stable and so the best suited for normalization of relative quantification gene expression studies. Several factors have an influence on the variability of control genes and may lead to differences between studies. In the same way that gene control expression may vary depending on whether the model is permanent (pMCAO) or transient (tMCAO) [27], different methodologies employed to perform the pMCAO, electrocautery or intraluminal filament, may have an influence on the gene expression. Sample collection and the control sample used are other factors which must be taken into consideration. Three different cerebral regions have been collected in each previous study: the whole of the infarcted cortex [21,22], the whole of the infarcted hemisphere [16] and the ischaemic penumbra cortex [23]. In our study we have collected the infarcted and peri-infarcted cortical area to analyse changes in gene expression in the affected region. With regards to the control sample there are also different possibilities: brain samples from sham or naive rats, or healthy contralateral hemispheres. We consider sham rats to be the best control because this control minimises bias due to surgery. In our opinion, the use of the healthy contralateral hemisphere may lead to erroneuos conclusions because studies have reported structural changes as well as changes in gene expression in the contralateral hemisphere after ischaemia [28-30]. Finally, the time course studied is also a relevant factor. We have validated Sdha, Ywhaz and Hprt as a suitable housekeeping genes for the pMCAO model conducting an analysis of the control situation and after ischaemia at frequent time intervals up to 72 hours, which vary from those used in earlier studies [16,21-23]. Considering all the variables that may influence gene expression, the validation of candidate reference genes in each individual experimental condition is crucial.

**Table 2:** Housekeeping gene validation in the pMCAO model

	**Harrison DC 2000**	**Medhurst AD 2000**	**Meldgaard M 2006**	**Tian YF 2007**	**Gubern C**
**Focal ischaemia model**	pMCAO intraluminal filament	pMCAO intraluminal filament	pMCAO electrocautery	pMCAO intraluminal filament	pMCAO electrocautery

**Animal**	Male Sprague-Dawley rats	Male Sprague-Dawley rats	Male SJL mice	Male Sprague-Dawley rats	Male Fisher rats

**Collected sample**	Whole infarcted cortex	Whole infarcted cortex	Whole infarcted hemisphere	Ischaemic penumbra cortex	Infarcted and peri-infarcted cortex region

**Time points**	Naive, 3, 6, 12 and 24 h (pMCAO and sham)	Contralateral hemisphere, 6 and 24 h	Sham, 30 min, 1, 2, 4, 6, 12, 24 and 48 h, 5 and 10d	Sham, 2 h, 24 h, 3, 7 and 21d	Sham, 30 min, 2, 6, 24, 48 and 72 h

**Real-Time PCR**	Custom TaqMan assays	Custom TaqMan assays	Custom TaqMan assays	SyberGreen	Inventoried TaqMan assays

**Method**	Standard curve	Standard curve	%mRNA = 100 × (1+E)Δ^CT^	2^-ΔC'T^	2^-ΔC'T^

**Statistical analyses**	ANOVA	Student's unpaired t-test	Kruskal-Wallis + Dunn's	ANOVA + Tukey	ANOVA + Dunnett's

**Suitable controls**	Ppia & Gapdh	Ppia & Gapdh	Hprt & Gapdh	Rpl13a & Ppia	Hprt, Sdha & Ywhaz

**Unsuitable controls**	β-actin			Gapdh, β-actin	Oaz1, Gapdh, Ppia, Rpl13a, B2m, Gusb, 18SrRNA

The geNorm [12] and NormFinder [11] algorithms are now widely used to determine the most stable reference genes from a set of candidates genes with invariable expression [26,31-34]. We have only used algorithms on genes identified as stable housekeeping genes in the in-vivo model in our analysis as the software requires a minimum of three genes for the analysis.

In order to check the value of the validation of endogenous controls, we have used different housekeeping genes analysed in this study to normalise the expression of cyclooxygenase 2 (Cox2) and Bcl2, genes which are known to be involved in cerebral ischaemia (data not shown). Cox2 is an inflammatory mediator gene which undergoes a significant increase due to ischaemia [35,36]. Bcl2 is a protooncogene that codifies an antiapoptotic protein, which has been described as participating in neuroprotective mechanisms in ischaemic stroke although changes in the expression are limited to an increase in the neurons within the peri-infarct region [37-39]. In the two cases we have observed differences in the results obtained when suitable and unsuitable control genes are used. These are even more critical when the differences in expression of the analysed gene are minor, as is the case with Bcl2, which has a different expression pattern depending on the housekeeping gene that is used.

## Conclusion

In designing any study into relative gene expression quantification it is necessary to perform a validation of the housekeeping genes used which takes into account the specific experimental conditions to be encountered and the time course to be employed.

We report a detailed investigation of the effects of experimental ischaemia on the expression of potential housekeeping genes including genes typically used and new candidates as internal controls in qRT-PCR assays. This analysis was facilitated by the sensitivity and efficiency of real-time quantitative PCR. We determined that Gapdh, Rpl13a and Ppia, which have previously been validated as housekeeping genes in quantitative expression studies with the pMCAO model, had significant differences in their expression in our samples collected at different time points. Our study confirms the suitability of Hprt as a housekeeping gene for this in-vivo model and identifies two new housekeeping genes, Sdha and Ywhaz, which are more stable than Hprt. We have also identified, to the best of our knowledge for the first time, reference genes (Rpl13a and Sdha) for the OGD in-vitro model using primary astrocyte cultures.

The study also highlights the importance of validating control genes for each individual study as gene expression is influenced by many different factors which can lead to mistaken conclusions.

## Methods

All procedures were approved by the Committee of Animal Care of the Universidad Complutense de Madrid, in accordance with the regulations of the European Union (86/609/CEE) and Spanish legislation (RD223/88).

### Reference gene selection

Candidate reference genes were selected from those most commonly used in the literature [14,24-26], a new candidate [10], and those previously described as suitable for pMCAO studies [16,21-23] (see Table 3).

**Table 3:** Candidate reference genes

**Gene Symbol**	**Gene Name**	TaqMan Assay Number*	**Genebank accession**
**Hprt**	*Hypoxanthine phosphoribosyltransferase*	Rn01527840_m1	NM_012583
**Gapdh**	*Glyceraldehyde-3-phosphate dehydrogenase*	Rn99999916_s1	NM_017008
**Oaz1**	*Ornithine decarboxylase antizyme 1*	Rn00821793_g1	NM_139081
**β2m**	*Beta-2 microglobulin*	Rn00560865_m1	NM_012512
**Ppia**	*Peptidyl-prolyl cis-trans isomerasa*	Rn00690933_m1	NM_017101
**Rpl13a**	*Ribosomal Protein L13A*	Rn00821946_g1	NM_173340
**18S**	*Eukaryotic 18S rRNA*	Hs99999901_s1	X03205.1
**Ywhaz**	*Tyrosine 3-monooxygenase*	Rn00755072_m1	NM_013011.3
**Sdha**	*Succinate dehydrogenase complex, subunit A*	Rn00590475_m1	NM_130428.1
**Gusb**	*Glucuronidase, beta*	Rn00566655_m1	NM_017015.2

*"_m" indicates an assay whose probe spans an exon junction and will not detect genomic DNA. **"_s" **indicates an assay whose probes and primers are designed within a single exon and, hence, will detect genomic DNA. **"_g" **indicates an assay that may detect genomic DNA as the assay probe and primers may also be within a single exon.

### In-vitro ischaemia

#### Rat astrocyte cultures

Primary astrocyte cultures were prepared from neonatal (P0) Wistar rat cortex, as previously described [40,41].

#### Exposure of astrocyte culture to oxygen-glucose deprivation (OGD)

Oxygen-glucose deprivation was performed as previously described [42]. Culture medium was replaced by a solution containing (in mM) NaCl (130), KCl (5.4), CaCl2 (1.8), NaHCO3 (26), MgCl2 (0.8), NaH2PO4 (1.18) and 2% FS bubbled with 95% N2/5% CO2 for OGD cells (OGD solution). OGD cells were transferred to an anaerobic chamber (Forma Scientific, Hucoa Erloss, Spain) containing a gas mixture of 95% N2/5% CO2 and humidified at 37°C, and maintained at a constant pressure of 0.15 bar. Time of exposure to OGD was 180 min. OGD was terminated by replacing the exposure medium with oxygenated MEM containing 0.6% glucose, 0.029% glutamine, 50 I.U./ml penicillin, 50 Ag/ml streptomycin, 10% FS (reperfusion medium) and returned to the normoxic incubator. Control cultures in a solution identical to OGD solution except that it contained glucose (33 mM; control solution) were kept in the normoxic incubator for the same time period as the OGD, and then incubation solution was replaced with the reperfusion buffer. Cultures were returned to the normoxic incubator until the end of the experiment.

#### Experimental groups

Astrocyte samples were collected from controls and from the OGD group at 0 minutes, 30 minutes, 2 hours, 6 hours, 24 hours and 48 hours after reperfusion (n = 5 in each group). Astrocytes were collected in RNA Protect Cell Reagent (Qiagen, Barcelona, Spain) and frozen at -80°C until RNA isolation was undertaken.

### In-vivo cerebral ischaemia

#### Permanent middle cerebral artery occlusion (pMCAO) in rats

Experiments were performed on male Fischer rats weighing 250–300 g. Rats were anaesthetised with 2.5% halothane in a mixture of 70% nitrogen/30% oxygen. Permanent focal cerebral ischemia was induced by ligature of the left common carotid artery (CCA) and occlusion of the ipsilateral distal middle cerebral artery (MCA) as previously described [41,42]. Briefly, for the CCA ligature, a midline ventral cervical incision was made, and the CCA was isolated and permanently occluded with a silk ligature. For the MCA occlusion, a 1-cm incision perpendicular to the line connecting the lateral canthus of the left eye and the external auditory canal was made to expose and retract the temporalis muscle. A 2-mm burr hole was drilled and the MCA was exposed by cutting and retracting the dura. The MCA was elevated and cauterised. Rats in which the MCA was exposed but not occluded served as sham-operated controls (SHAM). Following surgery, subjects were returned to their cages and allowed free access to water and food. The body temperature of animals was monitored throughout the experiment and was maintained at 37.5 ± 0.5°C using a heating pad.

#### Experimental groups

Brain were removed and cortical infarcted and peri-infarcted tissue was collected from controls and the pMCAO group at 30 minutes, 2 hours, 6 hours, 24 hours, 48 hours and 72 hours after artery occlusion (n = 6 in each group). Samples were quickly transferred to 1.5 mL tubes and frozen at -80°C until RNA isolation was undertaken.

### RNA extraction, quantification and retrotranscription

Cerebral cortex frozen samples were immediately transferred to QIAzol lysis reagent (Qiagen) and then homogenised using an Ultra-Turrax T25 homogeniser. RNA was isolated using the RNeasy^® ^Lipid Tissue Mini Kit (Qiagen) in accordance with the manufacturer's instructions and treated with DNase (RNase-free DNase Set, Qiagen) in order to remove any trace of genomic DNA. RNA was quantified spectrophotometrically (GeneQuant, Biochrom) at 260 nm (A_260_) and purity was estimated by an A_260_/A_280 _ratio > 1.8. The integrity, purity, and amount of RNA were verified by visualization of rRNAs after electrophoresis on agarose gel.

Total RNA was isolated from primary glial cultures using the RNeasy^® ^Plus Mini Kit (Qiagen) and total RNA concentration was measured by fluorescence using a Quant-iT RNA Assay Kit (Invitrogen, Barcelona, Spain). Manufacturers' instructions were followed in both cases.

2 μg of RNA from each cortex samples and 1 μg of RNA from astrocyte samples were reverse transcribed using a High Capacity cDNA Reverse Transcription Kit (Applied Biosystems, Barcelona, Spain) according to the manufacturer's instructions. Two independent RT reactions were performed for all samples.

### Real-time PCR

RT-Q-PCR reactions were carried out for all genes of interest in each sample using specific TaqMan^® ^Gene Expression Assays (Table 3) and an ABI PRISM^® ^7000 Instrument (Applied Biosystems). Each reaction was performed in a final volume of 25 μL containing 11.25 μL cDNA diluted with H_2_O, 1.25 μL TaqMan^® ^Gene Expression Assay and 12.5 μL of TaqMan^® ^Universal PCR Master Mix (Applied Biosystems). Amplifications were performed starting with a 2 min activation step for Amperase UNG at 50°C, 10 min template denaturation step at 95°C, followed by 40 cycles of 95°C for 15s and 60°C for 1 min. As we used TaqMan^® ^Gene Expression Assays validated with PCR efficiency near 100% for all of them, the expression of each housekeeping gene was obtained using the 2^-ΔC'T ^method, a modification of the 2^-ΔΔCt ^method described by K. Livak et al., where ΔC_t _(C_tTime X _- C_tControl_) and Control is the calibrator sample representing the 1× expression of each gene [13,24]. All samples were run in triplicate and average values were calculated. Two independent reverse transcriptions were tested for each gene.

As some of the Taqman Gene Expression assays employed are not cDNA specific, we confirmed no genomic DNA contamination of our samples using no-reverse transcription controls. A no-reverse transcription control is a reaction that has been prepared for reverse transcription with RNA and all the components of the retrotranscription kit except for reverse transcriptase.

### Statistical analysis

Data are mean ± standard error of the mean (SEM). A statistical test was applied to look for significant differences between experimental conditions for each candidate control gene in each experimental model. The variance equality hypothesis was confirmed for each gene in the two experimental models as Bartlett's test failed to reveal any significant variance with a confidence level of 95% (data not shown). A one-way analysis of variance (ANOVA) test was therefore conducted, followed by Dunnett's post-hoc analysis to compare each time set against the control group mean. A p value < 0.05 was considered statistically significant. Calculations were performed using the 11.0.0 version of SPSS software (SPSS Inc).

### Determination of reference gene expression stability

After determining which housekeeping genes had an invariable expression in the in-vivo model using 2^-ΔC'T ^method and ANOVA + Dunnett's post hoc analysis, we used two publicly available software tools, geNorm [12] and NormFinder [11], to analyse their gene expression stability. GeNorm calculates a gene-stability measure M, which is the average pairwise variation of a particular gene compared with all other candidate reference genes. A lower value of M indicates the greater stability of the reference gene. NormFinder estimates the overall expression variation of the candidate normalization genes and the variation between sample subgroups of the sample set using a model-based approach.

## Authors' contributions

CG performed all the experimental procedures, carried out the analysis and interpretation of data and was the primary author of the manuscript. OH assisted in obtaining and maintaining the primary astrocyte cultures as well as in performing OGD experimental work. RR assisted in RNA purification and in obtaining cDNA from cortex and astrocyte samples. JRM carried out all animal surgery. VGR assisted in animal surgery and tissue sampling. MAM, IL and JS participated in the discussion of data and critically reviewed the manuscript. JM participated in the conception and design of the study, the analysis and interpretation of data and helped to draft the manuscript. All authors read and approved the final manuscript.
